# Lateral gene transfer between prokaryotes and multicellular eukaryotes: ongoing and significant?

**DOI:** 10.1186/1741-7007-7-20

**Published:** 2009-05-05

**Authors:** Vera ID Ros, Gregory DD Hurst

**Affiliations:** 1 Department of Biology, Leidy Laboratories 326, University of Pennsylvania, 433 South University Avenue, Philadelphia, PA, 19104-6018, USA; 2 School of Biological Sciences, University of Liverpool, Crown Street, Liverpool, L69 7ZB, UK

## Abstract

The expansion of genome sequencing projects has produced accumulating evidence for lateral transfer of genes between prokaryotic and eukaryotic genomes. However, it remains controversial whether these genes are of functional importance in their recipient host. Nikoh and Nakabachi, in a recent paper in *BMC Biology*, take a first step and show that two genes of bacterial origin are highly expressed in the pea aphid *Acyrthosiphon pisum*. Active gene expression of transferred genes is supported by three other recent studies. Future studies should reveal whether functional proteins are produced and whether and how these are targeted to the appropriate compartment. We argue that the transfer of genes between host and symbiont may occasionally be of great evolutionary importance, particularly in the evolution of the symbiotic interaction itself.

## Commentary

Although lateral gene transfer (LGT) is known to play an important role in the evolution of prokaryotes and unicellular eukaryotes [1-3], lateral transfer between prokaryotes and multicellular eukaryotes has been more controversial. In recent years, evidence has accumulated for genes of prokaryotic origin – particularly bacterial symbiotic origin – within eukaryotic genomes. The examples have come from fractions (and even nearly complete copies) of the genome of the bacterial symbiont *Wolbachia *in the host nuclear genome [4-7]. However, there has been little evidence that the transferred copies of the genes are functional in the eukaryotic genome. For example, only very low expression levels have been found for some transferred genes, and this may represent no more than background noise [5,8]. Their dynamics seem to be similar to that of mitochondrial genes that have recently transferred to the nucleus (numts) – a balance of new copies appearing and their subsequent degradation associated with lack of function. However, recent studies have shown cases in which transferred prokaryotic genes are actively expressed in the eukaryotic recipient, a first step in demonstrating the full functionality of horizontally acquired genes in eukaryotes.

Nikoh and Nakabachi [9] show that the pea aphid *Acyrthosiphon pisum *seems to have acquired two genes from bacteria. These have probably been acquired independently from facultative secondary symbionts: one from *Wolbachia *or a close relative, the other from an undescribed bacterium. The authors further demonstrate that these genes are both highly expressed in the bacteriocytes, specialized cells that harbor the aphid's obligate primary symbiont *Buchnera aphidicola*. *Buchnera*, which has a strongly reduced genome, lacks both genes, whereas most other bacteria, including *Buchnera's *close free-living relatives, possess these genes. Both genes may be functionally essential to maintain *Buchnera *– making the nuclear inserted copy a strong candidate for being functionally active, with the product targeted to the endosymbiont. In addition, functionality is implied by the observation that the bacterial source is not currently present in the aphid – implying that the transfer is not recent – and pseudogenization would be expected in the absence of positive selection for function.

The aphid study [9] is one of several recent papers describing lateral transfer within symbiosis. Rumpho *et al. *[10] found evidence for LGT between two eukaryotes, the alga *Vaucheria litorea *and its predator, the sea slug *Elysia chlorotica*. By feeding on *V. litorea*, *E. chlorotica *obtains the algal plastids, which continue to photosynthesize for months in the sea slug. This is surprising, because the majority of proteins needed for photosynthesis are encoded on the algal nuclear genome. Rumpho *et al. *[10] now speculate that the sea slug might successfully maintain photosynthesizing chloroplasts because it has acquired essential genes by LGT from the algal genome, and they provide evidence for LGT of a nuclear gene (*psbO*, encoding a protein subunit of the photosystem II complex) from prey to predator. They also show that the gene is expressed in the sea slug.

Two other studies refer to an ancient LGT event between mosquitoes and the endosymbiont *Wolbachia pipientis *[11,12]. The transfer concerns genes encoding salivary gland surface (SGS) proteins of mosquitoes, which have a role in insect-*Plasmodium *interactions. Similar genes have been found in two of the six sequenced *Wolbachia *genomes. The function of the gene in *Wolbachia *is unknown, but it has diverged substantially from its mosquito equivalent, is not pseudogenized and is expressed. No equivalents in other prokaryotic or eukaryotic sequence databases have been found. The direction of transfer (from bacterium to mosquito or from mosquito to bacterium) remains disputed.

The above-mentioned cases probably represent relatively ancient transfers (two cases concern transfers from endosymbionts currently not infecting the host species) that lead to expressed genes in the recipient genome. Functionality of the protein product of the genes awaits confirmation. In two of the cases, the products function in a different compartment from where they are encoded (for example, being encoded in the host genome but putatively functioning in a symbiont or plastid) and establishment of functionality will require the demonstration both that the mRNA is translated and that the protein products are targeted to the appropriate compartment (the symbiont or plastid). Indeed, the targeting of these proteins is a potential barrier to the process of establishment of genes that have been transferred laterally. The protein must not only be expressed on transfer to the host, but must also move into the symbiont compartment to actually function. Gene transfer may be constrained for the same reasons as in the case of mitochondria: if compartmentalization of function (and thus the presence of the symbiont) is important, the major constraining factor for transfer is the ease of importability of proteins (targeting, import and assembly) into the compartment [1,13]. Hydrophobic proteins – which are difficult to translocate across membranes – notably remain encoded in the mitochondrial genome.

Notwithstanding formal establishment of functionality, one can ask what selective processes would drive the spread of the genes into their new compartment. For some cases, it may simply be a 'new function' that is selectively advantageous within that compartment. In others, the drive for the transferred gene seems to be complementing loss in another compartment. For the case of the predatory sea slug [10], the loss of the algal nuclear genome following predation makes the utility of genes that maintain a functional chloroplast clear. For the case of the aphid [9], the transferred gene could be driven into the population by the presence of a poorly performing or absent copy in the Buchnera genome. Buchnera, like other endosymbionts, has a genome that is greatly reduced in size compared with their free living relatives, resulting from pseudogenization and subsequent gene loss [14]. The transfer of a gene into the host from other microbial symbionts may have compensated for the deterioration of the gene in Buchnera, producing a selective advantage for the laterally transferred copy.

Although symbiont-host lateral transfer is both surprising and exciting at first sight, retrospectively, the presence of lateral transfer should have been expected – transfer of function to the nucleus has been a fundamental part of the evolution in the mitochondria-eukaryote symbiosis, and transfer of mitochondrial DNA genes to the nucleus that subsequently were pseudogenized (numts) is known to be both ongoing and common [15]. In this context, the finding of lateral transfer in more recent symbioses is less surprising, and the presence of functional copies of symbiont genes within the nuclear genome should therefore be expected – it was an integral part of early history of the mitochondria-eukaryote symbiosis and thus will be in these more recent symbioses. However, it is clear that the scope for such transfers is much wider than previously thought. First, lateral transfer is not limited to vertically transmitted symbionts, present in the germline – the alga is not vertically transmitted by the sea slug, but acquired through predation. Moreover, that vertical transmission is no prerequisite for LGT is evidenced by the recent finding of transfer of diverse bacterial and eukaryotic genes into bdelloid rotifers [16]. Second, transfers from bacteria to their host may be spreading by virtue of the phenotypic effect of the transferred genes in other bacterial symbionts. In the case of the aphid-*Buchnera *symbiosis, lateral transfer of non-*Buchnera *microbial genes to the host seems to be compensating for loss within the *Buchnera *genome.
